# Evaluation of News Communication Effect Based on Cognitive Neuroscience

**DOI:** 10.1515/tnsci-2019-0006

**Published:** 2019-04-23

**Authors:** Xiaozhu Chen, Fang Zhang

**Affiliations:** 1 School of Civil Engineering and Archetecture, Anhui University of Science and Technology, Huainan 232001, China; 2 School of Foreign Languages, Anhui University of Sicence and Technology, Huainan 232001, China

**Keywords:** News Communication Effect, Cognitive Neuroscience, Psychological cognitive mechanism

## Abstract

Based on the development of cognitive neuroscience and communication science at home and abroad, this study introduces the application of cognitive neuroscience experiments in communication researches at home and abroad, including the research and application of communication effects in advertising, picture, video, web design and animation culture. This study discusses the important significance of relevant frontier achievements to the development of news communication science. The rise of cognitive neuroscience provides a new perspective for understanding the psychological cognitive mechanism of the audience and optimizing the communication effect of service media. On the basis of introducing the status quo of cognitive neuroscience in audience rating evaluation of news broadcast, this study introduces the advantages and functions of this method in audience rating evaluation of news broadcast with eye movement experiment as an example, which provides a new method and empirical case for seeking audience rating evaluation of news broadcast.

## 1 Introduction

**Figure 1:**
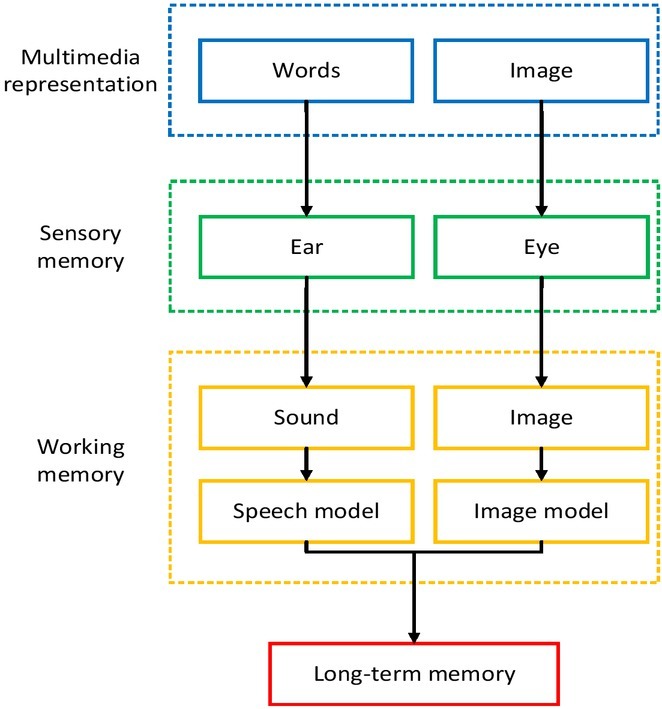
The cognitive theory model in news communication

Human brain is the physiological basis of human thought, emotion, imagination and behaviour ^[1, 2, 3]^, and its operation law is a pass that human beings can’t avoid in understanding themselves. Due to the lack of reliable experimental means, the traditional humanistic social science has been limited to subjective observation, introspection, as well as text analysis, questionnaires and interviews based on observation and introspection in terms of studying human cognition. The study process and conclusions are inevitably influenced by subjective factors. In addition, most of these studies focus on the level of conscious mind but pay little attention to sub-consciousness factors because of the limited research means. However, it is the sub-consciousness factors that have important influence on people’s emotion, thought and behaviour ^[4]^. With the continuous enrichment of relevant achievements, it is expected to form a new paradigm of news communication study. The cognitive theoretical model of news communication is shown in Figure 1.

The market evaluation of news broadcast has always been an important issue in media effect communication study. At present, the comprehensive news broadcast evaluation system is generally divided into three indexes: objective evaluation index, subjective evaluation index and income index ^[5]^. The objective evaluation index evaluates from the level of “quantity” of communication effect, and the audience rating is one of the objective evaluation indexes widely used in program quality evaluation. The subjective evaluation index evaluates from the level of “quality” of communication effect. At present, the subjective evaluation index commonly used in news broadcast is mainly obtained by expert scoring, leader scoring and audience scoring. The income index evaluates the communication value of programs in the advertising market from the level of two selling behaviours of news broadcast, and its data comes from the input-output calculation of program and the bearing and return of program advertisement. Among the three evaluation indexes, the objective audience rating index and the subjective scoring index are the two types of communication effect evaluation indexes that the audience is more concerned about. However, in reality, the data of objective audience rating are often inconsistent with the subjective satisfaction scoring results ^[6]^. Some TV programs and TV play series are highly rated by experts before they are broadcast, but the actual audience ratings after they are broadcast are not ideal. Some TV programs and TV play series are unwanted before they are broadcast, but their audience ratings after they are broadcast are rising. The contradiction between “applauding” and “drawing well” is worth thinking about.

This paper introduces the application of cognitive neuroscience experiments in domestic and international communication research, including the research and application of communication effects in advertising, pictures, video, web design and other fields. After that, it discusses the significance of related frontier achievements to the development of journalism and communication. Based on the introduction of the status quo of cognitive neuroscience and the evaluation of news broadcast ratings, this paper introduces the advantages and functions of this method in the evaluation of news broadcast ratings, and provides a new method.

## 2 Foundation of Cognitive Neuroscience

### 2.1 “Third Dimension” of news dissemination

The first dimension medium is the most basic and most critical of all communication activities, and it is the prototype of the latter two dimensions. The multi-level communication phenomenon of the first dimension media is still reflected in the print culture and electronic culture. The digital media once again possesses the characteristics of interaction and diversified communication modes in interpersonal communication. In addition, the human body is a key measure of future communication. The “metaphor we rely on for survival” depends first and foremost on the human body. The second dimension of the mass media makes up for the shortcomings of the first dimension of the media, extends the body, achieves one-to-one replication, storage and presentation of specific texts, and also expands the diffusion potential of information, enabling humans to Information is obtained across time and space, and is not affected by the presence or absence of participants and the amount of language. As a third dimension, digital media integration links all media, integrating text, images, and sound into many existing expression types, and re-mediating by reshaping the possibilities of each medium in a new online context. , gave birth to one-on-one, one-to-many and many-to-many network communication. Thus, there is a mutual influence, mutual absorption, and mutual adjustment between the media of the three dimensions.

The emergence of the medium of the third dimension has led to changes in the public sphere, the boundaries between the social sphere and the private sphere have become increasingly blurred, and the political public sphere and the cultural public sphere have become increasingly infiltrated. At the same time, in the public domain of networking, the interaction of social actors with each other is strengthened. For example, mobile media spans time and space, greatly expanding the availability of information and engaging it in the socialization of the self and the institutionalization of society. Therefore, the public organization of citizens has re-established its third force beyond the state and the market, and the political expansion of representation and communication is an interactive politics. It can be seen from the above three angles that the Internet technology makes the media characteristics of the media gradually disappear, and the position of the person gradually becomes prominent. Human beings

are the dominant factor in the development and integration of media. It is precisely the human drive for communication and communication that can be changed as the dissemination of social practice. The media that is endowed by human beings can be integrated and developed.

**Figure 2:**
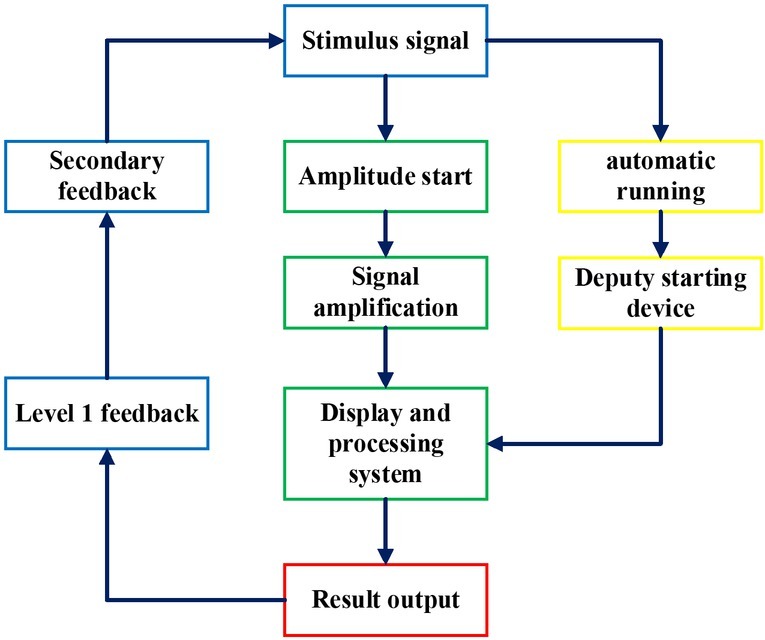
The structure of cognitive neuroscience

The target of cognitive neuroscience is to open the black box of human brain thinking and explore the material basis of human cognitive activities. It has two direct sources of disciplines, namely cognitive neuropsychology and neuroscience. One of the challenges faced by human science is to figure out the relationship between consciousness and brain. Since ancient Greek and the pre-Qin period in China, there has been a preliminary thinking. However, exploring the relationship between the mind and the brain in a scientific sense began with the study of different cognitive deficits caused by damage to different parts of the brain in the 19th century ^[7]^. In the middle of the 20th century, cognitive science began to systematically study perception, attention, memory, action, language, thinking, decision-making, motivation, emotional process and structure of human and animal. On the basis of these problems, researchers gathering psychology, linguistics, anthropology, computer science, neuroscience and other basic sciences realize a long-span cross and fusion of disciplines in the history of science ^[8]^. The structure of the cognitive neural network is shown in Figure 2.

This “third dimension” is captured by describing the sub-consciousness of the subjects through measurement of computer, eye movement or skin conductance. Such a study relies on what psychological and physiological responses they claim to have rather than their conscious responses. The uninterrupted data are obtained from physiological test when they watch the news broadcast.

Although the introduction of the experimental means of cognitive neuroscience into news communication science has only just begun, the achieved results are encouraging, and some conclusions may even have a subversive impact on communication science ^[9]^. This study analyses the mathematical modelling of cognitive neuroscience.

### 2.2 Mathematical models of cognitive neuroscience

A motion sequence M is given. When calculating the histogram of psychological response of the user, we first averagely divide [0,π ]into intervals, and the size of L is determined by the following formula.

(1) $$L=\left[\log_{2}N+1\right]$$

Where, N is the number of video sequence, and a boundary is formed after dividing [0,π] into intervals, as shown in the following formula.

(2) $$s_{i}=\frac{\pi }{L}*i,i=0,\ldots ,L$$

The divided interval is shown in the following formula:

(3) $$I_{l}=\left[s_{l-1},s_{l}\right)$$

The psychological behaviour partitioning algorithm based on PPCA realizes the behaviour partition by constructing Gaussian model with the data captured psychological response. The psychological behaviour partitioning algorithm based on PPCA has good partitioning performance. The following is a simple introduction to the behaviour partitioning algorithm based on PPCA ^[7]^.

The singular value decomposition (SVD) method is used to reduce the dimension of captured data of news broadcast videos ^[10]^.

(4) $$D=USV^{T}$$

By projecting all the lean in a news broadcast cast video sequence to this optimal subspace, all singular values other than the r dimension are discarded. The probabilistic principal component analysis estimates the Gaussian distribution by taking the extracted singular

values outside the principal component space as noise.

(5) $$\sigma^{2}=\frac{1}{q-r}\sum_{i}\sigma_{i}^{2}$$

When the behaviour partition is performed on the captured data, a Gaussian distribution is constructed for the 1st frame to the *k*-th frame of the news broadcast video sequence, where the mean value of the Gaussian distribution is obtained by the following formula.

(6) $$\bar{a}=\frac{1}{k}\sum_{i}a_{i}$$

The covariance matrix of the Gaussian distribution may be expressed as the following formula,

(7) $$W=V_{r}{\left(S_{r}^{2}-\sigma^{2}I\right)}^{1/2}$$

(8) $$C=\frac{1}{n-1}\left(WW^{T}+\sigma^{2}I\right)=\frac{1}{n-1}VS^{2}V^{T}$$

### Experimental analysis

Cognitive neuroscience is characterized by a multidisciplinary, multi-level, multilevel intersection. It combines behaviour, cognition and brain mechanism, trying to comprehensively expound human and animal perceptions of objects and forms from the macro level of molecules, synapses, neurons and other macro levels of system, whole brain and behaviour of the use of language, memory information, information processing process and its neural mechanisms in decision making.

The research approach of neuroscience mainly focuses on the four quadrants of information processing of human brain. To understand the division of these four quadrants

is instructive to explore the possibility of combining neuroscience with microscopic instantaneous transmission effect. First, this study explores three different eye movements, as shown in Table 1.

**Table 1:** Three basic forms of eye movement

Basic form of eye movement	Definition
Gaze	The fixation refers to the activity of the line of sight during which the central fossa of the eye is aimed at an object for more than 100ms, during which time the object to be gazed is imaged on the central fossa to obtain a more complete processing to form a clear image. When looking at the eye, the eye is not absolutely still. The eyeball constantly makes extremely fine vibrations to see the object. Its amplitude is generally less than 1°.
Eye-jumping	Saccade is a fast jumping activity between the gaze points of both eyes. The viewing angle is 1°-40°, the duration is 30ms-120ms or more, and the maximum moving speed is 400°-600°/S. During the saccadic process, almost no information can be obtained because of its speed.
Follow movement	Follow-up exercise is the process in which the eyeball moves slowly to observe the motion of the observing object as it moves, and the motion speed is about 1°-30°/s. It is often accompanied by a sip, and most people fall into this category when reading normally.

Neuroscience refers to the science of seeking to explain the biological mechanisms of mental activity, namely the mechanisms of cell biology and molecular biology. Neuroscience seeks to understand how neural circuits assembled during development experience the world around them, how they perform behaviours, and how they recover their perceptions from memory. Once recovered, they can also contribute to the memory of perception. Neuroscience also seeks to understand the biological foundations that support our emotional life, how emotions can change our minds, and why depression, arrogance, schizophrenia, and Alzheimer are when the regulation of emotions, thoughts, and movements is distorted by Symptoms and other diseases.

The research path of neuroscience mainly focuses on the four quadrants of human brain to information processing. To understand the division of these four quadrants is instructive to explore the possibility of the combination of neuroscience and microscopic instantaneous transmission effect. These four methods are specifically shown in Table 2.

**Table 2:** The four main types of information processing in the human brain

Features	Cognitive process	Emotional process
Controlled processing	Type I	Type II
Ccontinuously		
Need to work hard		
Actively evoked		
The process that can be clearly detected and recalled		
Automatic processing	Type III	Type IV
At the same time		
Do not need to work hard		
Reflective		
Can’t detect it by yourself		

**Figure 3:**
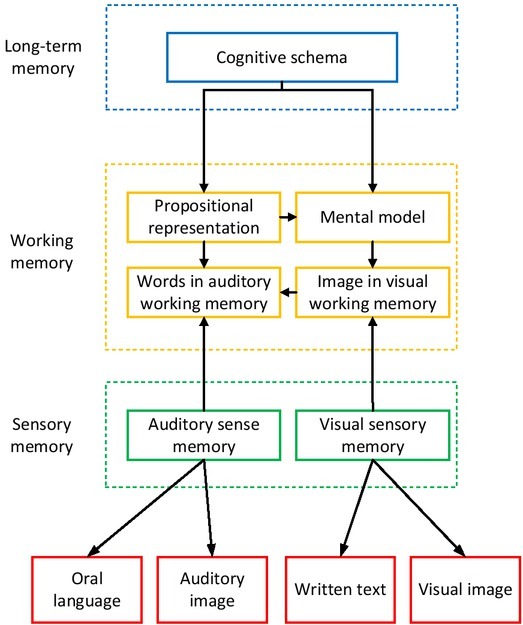
News understanding integration model

From a physiological point of view, the automatic processing process and controlled processing process can be roughly identified and classified from the parts of the brain they produce. With the conclusion and means of neuroscience, we may distinguish the mechanisms by which the instantaneous transmission effect of information is formed. As shown in Table 2, the second dimension is the partition between the emotional process and the cognitive process, which is quite common in modern psychology. The emotional process leads to the behaviour of “achieving” or “avoiding”. On the contrary, the cognitive process is to answer the question of “right or wrong”. It is difficult for the simple cognitive process to directly cause the change of behaviour, so cognitive process needs to act on the emotion system to influence the behaviour. The integrated model of news understanding is shown in Figure 3.

Table 3 is the ratio of short-term memory effect (300-500ms) between embedded advertisement and hard advertisement with different implantation levels in news broadcast. The following mean and variance show the range of effects of different embedded advertisements, which can be used as the basis of pricing research.

**Table 3:** Short-term memory effect of implanted advertisements and hard advertisements with different degree of implantation

Degree of implantation	Implantation means	Effect ratio with hard advertisement (M)
Shallow implant	Simple exposure	1．1±0．9
Moderate implantation	Discourse	1．2±1．0
Deep implantation	Episode implantation	1．9±0．9
	Comprehensive three different levels of implantation	1．3±1．1
	Comprehensive European and American standards	2．2±2．0

The experimental result shows that the ranking of eye movement is consistent with that of the news broadcast, and has significant correlation. First, the Spearman rank correlation is used to analyse the correlation between the number of fixation points and the audience rating, and the result shows that the correlation between the two is significant (r=1, p=0＜0.01). Secondly, there is a significant correlation between the time before the first fixation and the audience rating by Spearman rank correlation (r=-1, p=0＜0.001), as shown in Table 4.

**Table 4:** The time before the first gaze, the number of fixations and the rating analysis table

		Time before first sight	The number of fixations	Ratings
News ratings	Correlation coefficient	-1.23	1.34	1.00
	Sig. (two sides)	0	0	0
	N	3	3	3

From the variance analysis of the number of fixation points in all the selected plays, it is found that there is a significant difference between the three TV play series (F=12.341，p=0＜0.001). This shows that the eye movement data can well distinguish the difference of attention between different plays, which has accuracy in analysing the fine discrimination in different TV play ranking. The objective and accurate data are feasible in application. The specific analysis result is shown in Table 5.

**Table 5:** Variance analysis table for the number of fixations

	sum of square	df	Mean square	F	Significant
Between groups	12112. 224	2	6056. 11	12. 341	0.00
In groups	45639. 148	93	490. 744		
Total	57751. 372	95			

**Figure 4:**
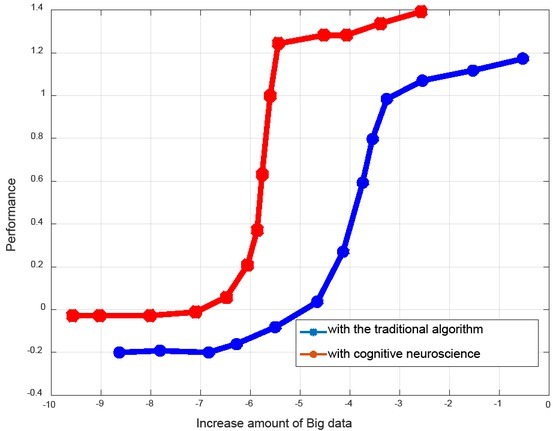
System performance

Finally, we predict the audience rating of news broadcast by analysing the traditional method and the method based on cognitive neuroscience proposed in this study, and the prediction accuracy is shown in Figure 4. It can be seen that the method based on cognitive neuroscience has better performance.

## Conclusions

As a new thing, new media has a tremendous impact on the development of traditional media. The survival and development of traditional media faces enormous challenges. First, the loss of traditional media audience is serious, and social benefits have fallen sharply. The latest research shows that many people have lost interest in traditional media, TV ratings, radio listening rates, newspaper purchase rates have fallen rapidly, and new media users have risen sharply. By the end of 2016, China’s netizens reached 73.1 billion, with a total of 42.99 million new netizens. The Internet penetration rate was 532%. The size of Chinese netizens has already reached the total population of Europe. Second, the traditional media market share has been continuously reduced, and economic benefits have fallen sharply. The new media has the advantages of employing mechanism, flexible capital operation and low cost. It has obtained considerable economic benefits while expanding its influence.

Under the condition of new media technology and global integration, the communication science is facing its own adjustment and theoretical construction. The introduction of cognitive neuroscience will be beneficial to the exploration of the dialogue between disciplines and the internal mechanism of communication. It is obviously not enough to use the ideas and methods of cognitive neuroscience alone. On the one hand, any means of research has its limitations and cognitive neuroscience can’t cover all the problems of news communication science. On the other hand, cognitive neuroscience is still in the exploration stage where the related theories and methods need to be further improved, and a series of important problems need to be further explored. We use the cognitive neuroscience for reference mainly in order to provide a new theoretical perspective and means for the audience research, media research and effect research of the current news communication science with its “intuitive human brain” method. In this way, we can try to establish a new paradigm for the basic theory construction and practical application of news communication science.
